# Efficient construction of a linkage map and haplotypes for *Mentha suaveolens* using sequence capture

**DOI:** 10.1093/g3journal/jkab232

**Published:** 2021-07-14

**Authors:** Helen Tsai, Nestor Kippes, Alana Firl, Meric Lieberman, Luca Comai, Isabelle M Henry

**Affiliations:** Department of Plant Biology and Genome Center, University of California, Davis, Davis, CA 95616, USA

**Keywords:** sequence capture, linkage map, *Mentha*, mapping-by-sequencing, method

## Abstract

The sustainability of many crops is hindered by the lack of genomic resources and a poor understanding of natural genetic diversity. Particularly, application of modern breeding requires high-density linkage maps that are integrated into a highly contiguous reference genome. Here, we present a rapid method for deriving haplotypes and developing linkage maps, and its application to *Mentha suaveolens*, one of the diploid progenitors of cultivated mints. Using sequence-capture via DNA hybridization to target single nucleotide polymorphisms (SNPs), we successfully genotyped ∼5000 SNPs within the genome of >400 individuals derived from a self cross. After stringent quality control, and identification of nonredundant SNPs, 1919 informative SNPs were retained for linkage map construction. The resulting linkage map defined a total genetic space of 942.17 cM divided among 12 linkage groups, ranging from 56.32 to 122.61 cM in length. The linkage map is in good agreement with pseudomolecules from our preliminary genome assembly, proving this resource effective for the correction and validation of the reference genome. We discuss the advantages of this method for the rapid creation of linkage maps.

## Introduction

With the decreasing cost of sequencing, *de novo* genome assemblies are being developed for a multitude of species. The successful development of an accurate genome assembly depends on intrinsic factors, such as genomic complexity, heterozygosity, and ploidy level, but also the technology used for the assembly.

Despite the many advances in sequencing technologies, it is difficult and expensive to sequence entire plant chromosomes in one continuous read (Dominguez Del Angel *et al.* 2018; Sohn and Nam 2018; Rice and Green 2019). Instead, sequences are obtained in fragments, which need to be combined by collapsing shared regions. A major hindrance to genome assembly is the ubiquitous presence of repeated sequences, which are challenging to piece together in the correct order. This can be further complicated by the presence of extensive heterozygosity. As a consequence, draft genomes still face gaps in assembly, fragmentation, or misassembly of sequences (Dominguez Del Angel *et al.* 2018; Sohn and Nam 2018; Rice and Green 2019). While the assembly can be improved via specialized sequencing and computational methods, many are expensive and challenging to apply.

An effective and well-established method for assembly validation is the use of linkage maps. The first genetic linkage map is over 100 years old (Sturtevant 1913). More recently, high-density linkage maps composed of thousands of markers have been used to correct and validate numerous genome assemblies in plant species such as *Brachypodium* *distachyon* (Febrer *et al.* 2010), cotton (Wang *et al.* 2015), maize (Wei *et al.* 2009), flax (You *et al.* 2018), wheat (Ariyadasa *et al.* 2014), in fungi such as *Gibberella zeae* (Lee *et al.* 2008), and in animals such as fugu (Kai *et al.* 2011). In addition to assisting genome assembly, linkage maps can provide interesting insights into a genome. For example, high recombination regions (hotspots) are typically gene-rich, while low recombination regions (coldspots) are typically transposable elements-rich, and consist of heterochromatin (Lichten and Goldman 1995; Gill *et al.* 1996; Shen *et al.* 2017). Linkage maps can also help provide insights into the genomic diversity and evolutionary history of important crops (Mahoney *et al.* 2016). Furthermore, the development of linkage maps can detect segregation distortion, enable functional genetic studies, and facilitate breeding, such as by the mapping of agronomic quantitative trait locus (QTL) (Zhang *et al.* 2010; Cui *et al.* 2015).

Linkage maps provide an independent source of data for genome assembly validation because loci are mapped based on recombination, avoiding the problems associated with sequence-based assembly. Linkage mapping is based on the principle that close syntenic loci are more likely to be co-inherited than distant ones (Morgan 1911; Sturtevant *et al.* 1919). While loci separated by 50 or more map units are unlinked, gaps of this size are exceedingly rare. Accordingly, we expect that genetic and physical mapping will place markers in the same groups—linkage groups or chromosomes, respectively (Punnett 1927), and in the same order. Therefore, genetic mapping can join scaffolds that appear separated and identify errors in assembled sequences.

The resolution of a linkage map, and thus the extent to which it can validate and correct small regions of a physical map, depends on the size of the mapping population and the number of markers targeted. Markers can be genotyped by whole-genome resequencing, but deep coverage is costly and generates excess data. Low coverage whole-genome resequencing is more affordable, but results in decreased resolution in heterozygous sites, and thus higher computation requirements and higher error rates. Genome complexity reduction techniques reduce the genome space sampled to a much smaller size but do not target specific sites (Miller *et al.* 2007; Baird *et al.* 2008; Davey *et al.* 2011; Elshire *et al.* 2011; Ali *et al.* 2016).

Here, we chose to specifically target single nucleotide polymorphism (SNP) markers evenly dispersed throughout the genome, and identified by sequencing of the parental genome. Specifically, we employed a “solution-capture” method that uses RNA baits (probes) complementary to target sites, in order to selectively target DNA fragments surrounding SNPs. The RNA probes are biotinylated which allows for the isolation of fragment-probe heteroduplexes using magnetic streptavidin beads (Gnirke *et al.* 2009). The benefits of this approach are (i**)** ability to target specific loci (specificity), (ii**)** robustness despite polymorphic target-regions, (iii**)** relatively uniform and reliable high coverage sequencing of the targeted positions, and (iv**)** cost-effectiveness for the sequencing phase and computational analysis (Henry *et al.* 2014; Neves *et al.* 2014; Hugall *et al.* 2016; Tennessen *et al.* 2017).

Mint (*Mentha* spp.) is a species with poor genomic resources. Mint is herbaceous and perennial, and cultivated for its aromatic essential oils. Improvement of cultivated mints has been hampered by the fact that they are polyploid hybrids, clonally propagated, and usually sterile. Two diploid species, *Mentha* *suaveolens* and *Mentha* *longifolia*, have been identified as progenitors of the polyploid cultivated mints (Harley and Brighton 1977; Gobert *et al.* 2002), and their characterization is more straightforward. A reference genome for *M. longifolia* was published a few years ago, although with low contiguity (Vining *et al.* 2017). A physical map of a clone from the other diploid progenitor of cultivated mints, *M. suaveolens*, was previously assembled (Firl et al., manuscript in preparation). This accession was chosen because it is self-fertile and previous cytological observations revealed it to be diploid with 24 chromosomes (Chambers and Hummer 1994). To validate this assembly, and provide an additional functional genetic tool, we constructed the first high-density linkage map for mint on an S1 population obtained by selfing this single parental clone, using a high-throughput hybridization-based SNP capture approach. After strict filters, we successfully genotyped 83% of the targeted SNPs. After removal of SNPs that were not directly informative for this set of recombinants, 1919 informative SNPs were retained for linkage map construction. The resulting linkage map defined 12 linkage groups in good agreement with the 12 assembled scaffolds, proving this resource effective for the correction and validation of the reference, as well as for providing a valuable functional genomic resource for future investigations.

## Materials and methods

### Plant materials

The *M. suaveolens* population described in this study was based on the line *M. suaveolens* 10021 (Pl 557898 or CMEN 13) obtained from the USDA Mint Germplasm Collection (Corvallis, OR, USA). This line was previously described as diploid (*n* = 24) and fertile (Chambers and Hummer 1994). To develop the mapping population, this accession was grown to maturity under greenhouse conditions (22_**°**_C and natural light), the inflorescences were bagged before anthesis to favor self-fertilization. When completely dried, flowers from different inflorescences were harvested and the seeds cleaned and inspected under a dissection microscope. Seeds were subject to a 15 minutes treatment with 12% bleach, under gentle agitation and two washes with sterile water, before a stratification treatment of 10 days in plates containing 0.5X MS agar at 4_**°**_C in the dark. After stratification, seeds were moved to *in vitro* cups with 0.5X MS agar and maintained at 21_**°**_C and artificial light. Seedlings were transferred to soil into 72 cells seedling trays and grown under greenhouse conditions until enough tissue from each plant could be harvested for DNA extraction.

### Capture probe design

Probes were designed to target SNP positions that were identified as heterozygous in the parental line CMEN13 after mapping of Illumina PE150 reads from CMEN13 onto our draft genome assembly of the same accession (A. Firl, manuscript in preparation). SNP selection was based on the following criteria. First, SNPs must display confident biallelic heterozygous counts of alleles in the parental line CMEN13. Specifically, positions were retained only if they exhibited >30X read coverage and were biallelic with at least 40% of each allele. Second, SNPs were selected such that at least one SNP was retained for every 100,000-kb. Third, SNPs located within exons were prioritized. In some cases, no SNP could be recovered for a given scaffold because they were short, contained repetitive sequences, or had low heterozygosity. Once SNPs were selected, the probes were designed and further evaluated by the manufacturer, Arbor Biosciences for final selection. The probe sequences were compared to the reference sequence under a certain primer melting temperature (Tm) using BLAST, and no more than five hits outside the target region were allowed. An initial set of 4884 single-copy SNP markers were targeted using a three overlapping oligonucleotide probe strategy: one flanking each side of the SNP position and one centered on it. Each probe hybridizes to a 99-bp region and the combined triplet spans 140-bp. The resulting list of pseudomolecules and number of corresponding SNPs targeted are listed in Table 1. The probes sequences are available in Supplementary Table S2.

**Table 1:** Summary of pseudomolecule statistics

Type	Assembly name	Size (Mbp)	% Genome	No. markers	Markers retained
Scaffold	S01	62.45	11.64	464	168
	S02	51.27	9.56	430	141
	S03	51.75	9.65	491	194
	S04	49.09	9.15	510	211
	S05	46.64	8.70	459	174
	S06	44.69	8.33	323	137
	S07	44.57	8.31	375	149
	S08	38.13	7.11	335	133
	S09	37.15	6.93	328	125
	S10	40.48	7.55	428	194
	S11	31.60	5.89	381	139
	S12	30.25	5.64	322	130
Contig	X1059	0.02	0.004	1	0
	X1121	0.01	0.003	1	0
	X1124	0.01	0.003	1	0
	X1181	0.01	0.001	1	1
	X233	0.12	0.023	1	0
	X321	0.45	0.084	1	0
	X368	0.05	0.009	1	0
	X394_1	1.35	0.251	22	18
	X394_2	0.94	0.175	6	4
	X394_3	0.12	0.023	2	1
	X713	0.01	0.001	1	0
Scaffold		528.07	98.457	4,846	1,895
Targeted Contig		3.10	0.578	38	24
Not Targeted Contig		5.17	0.964	——	——

Targeted scaffolds and unintegrated contigs and their respective size (Mbp), percentage of the genome, number of SNPs targeted, and number of SNPs retained for the final linkage map.

### Sequencing library preparation

Fresh leaf tissue was harvested and frozen for genomic DNA extraction. An internal protocol, similar to Qiagen DNeasy Plant Kit, was used for the extraction of DNA. Sample quality was assessed using agarose gel electrophoresis and quantified using fluorescent dye, SYBR Green I. Illumina genomic library preparation input was 0.5–1_** µ**_g in order to reduce the number of amplification cycles to help minimize polymerase chain reaction duplicates, and improve library complexity (Head *et al.* 2014). For this reason, samples with less than 0.5_** µ**_g starting input were not included.

Fragmentation was performed using a Covaris E220 sonicator at the factory settings for 300 bp in 130_** µ**_l. The fragmented DNA was purified using in-house magnetic beads (Rohland and Reich 2012). Libraries were then constructed using the Roche KAPA Hyper Kit (catalog #KK8514) following the manufacturer’s instructions. Enrichment was performed with 6 cycles. Libraries were quantified using SYBR Green I and combined in various pool sizes (from 23 to 48 libraries per pool) in equimolar amounts for capture. Pools were bead-cleaned and size-selected with agarose gel-extraction targeting 200–500 bp. Samples were further concentrated with magnetic bead cleanup and 500 ng of input was eluted to 7_** µ**_l for capture.

Capture libraries were made using the MyBaits Hybridization Capture for Targeted NGS (Arbor Biosciences) following the manufacturer’s instructions with a few exceptions. The recommended hybridization temperature is 65_**°**_C, but prior experience had demonstrated that 63_**°**_C for 16 hours increased capture yield without compromising specificity. We also substituted the supplied Block C and Block O with Roche EZ Developer Mix, plant repeat blockers (# 06684335001). Capture enrichment was done for 10–12 cycles. Final capture libraries were purified with magnetic beads, quantified with the Qubit dsDNA HS Assay Kit (Invitrogen), and pooled in equimolar amounts. Pooled library quality was assessed using the Agilent Bioanalyzer 2100 DNA Kit and qPCR. A subset of 143 libraries were sequenced in one lane of PE150 Illumina HiSeq4000 sequencer. The remaining 296 libraries were sequenced in approximately 28 percent of a lane of PE150 Illumina NovaSeq sequencer. Quality assessment and sequencing were carried out by the DNA Technologies and Expression Analysis Core at the UC Davis Genome Center.

### Read processing

**Figure 1:**
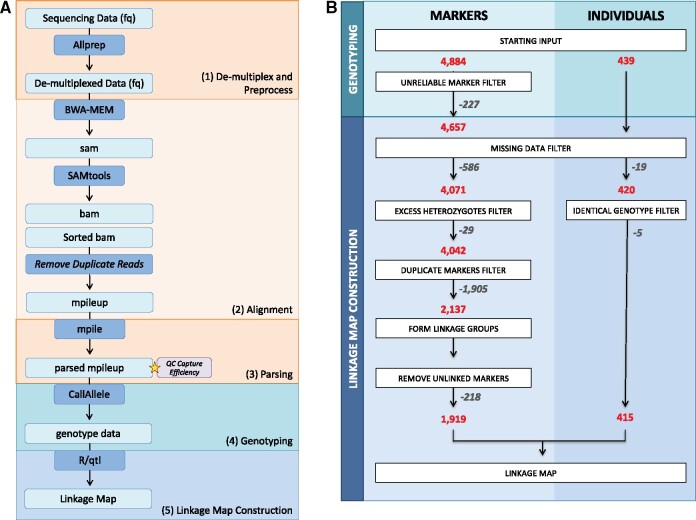
Overview of the data processing steps performed. (A) Flow diagram of the steps from the processing of raw sequencing reads to the start of linkage map construction. (B) Diagram of the filtering processes, including the number of individuals and markers removed and retained at each step.

A flowchart outlining the processing steps prior to linkage map construction is found in Figure 1A. The general steps were: (i) read quality filtering and de-multiplexing, (ii) alignment to the reference genome, (iii) parsing of the alignment file for genotyping, and (iv) genotyping.

First, raw sequencing reads were processed using a custom Python script called Allprep.py (https://github.com/Comai-Lab/allprep). This script uses raw reads as input and performs the following steps: de-multiplexing, trimming for quality (threshold greater or equal to 20 average Phred sequencing quality score over a 5-bp sliding window), filtering for minimal length (threshold 35-bp), and trimming for ambiguous N bases. Second, processed reads were aligned to the current version of the *M. suaveolens* genome (unpublished Firl *et al.*) using the Burrows-Wheeler Alignment (BWA) tool and default parameters (Li and Durbin 2009). Duplicate reads were removed and the data were then concatenated into a single mpileup.txt file using SAMtools (Li *et al.* 2009). Third, this mpileup file was parsed using a custom Python script mpileup-parser.py (https://github.com/Comai-Lab/mpileup-tools). The parser outputs the percentages of each base call at each position and each sample for genotyping. Fourth, each individual was genotyped at each SNP position using a custom Python script (CallAllelesAB-vHT5.py, Supplementary Data S1). Briefly, for each individual and each position, read coverage was assessed and positions that do not fall within the acceptable coverage range, 20X to 1000X, were assigned as N/A. Furthermore, SNP positions were only retained if at least 20% at the libraries exhibited acceptable coverage. Finally, genotypes were called A or B if only one allele of the two parental alleles was observed, or if both alleles were present but one represented less than 5% of the read calls. If both parental alleles were observed and they each represented at least 30% of the read calls, the genotype was called heterozygous (AB). Intermediate situations were assigned as N/A. After genotyping, we formatted our data for the F2 intercross analysis of R/qtl (Broman and Sen 2009).

Capture efficiency was assessed with an internal Python script by comparing the average coverage of the regions targeted by the probes to the average coverage over the rest of the genome. Visual assessment was conducted to confirm reads mapped to expected target regions using Integrative Genomics Viewer (IGV) (Thorvaldsdóttir *et al.* 2013).

### Marker filtering

A flowchart outlining the marker and individual filtering process is found in Figure 1B. To derive the threshold for filtering individuals with excessive missing data, we looked for outliers in the distribution of numbers of typed markers per individual. For our dataset, the threshold was set at 3500 markers. The threshold for filtering markers that do not have sufficient numbers of genotyped individuals was set at 300. The R/qtl function *comparegeno()* was used to identify unusually similar individuals, indicative of double-sampling. Pairs of individuals with 90% or more similarity were flagged, and one from each pair was removed from analysis. Markers with segregation distortion were retained except for ones with greater than 90% heterozygous calls. The function *findDupMarkers()* was used to identify markers with identical genotypes, with the parameter *exact.only=FALSE*. If multiple markers were found to have identical genotypes, the marker with the least amount of missing data was prioritized. Markers were dropped using the *drop.markers()* function.

### Phase unknown dataset

Markers were phased using a strategy that closely follows the method described by Gadau *et al.* (2001). Shortly, we used the following steps: (i**)** For each marker, a second artificial “mirror” marker, with the complementary genotype, was introduced into the dataset. For example, if the observed genotype for four individuals at a marker was AAHB, then the artificial marker would be BBHA for the same four individuals. In effect, this doubled the size of the marker set. (ii**)** The R/qtl function *formLinkageGroups()* grouped markers into inferred linkage groups based on pairwise linkage information, recombination fraction (RF) and logarithm-of-odds (LOD). Linkage groups appeared in “mirror” pairs with complementary genotypes. We found that the parameters *max.rf* = 0.05 and *min.lod* = 10 produced 12 pairs (24 total) of linkage groups that cumulatively contained the vast majority of the markers (90%). Each linkage group pair was checked to confirm that they contained the same number of markers in the same order. (iii**)** Since “mirror” pairs contain the same but complementary information, keeping both members of the pairs would be redundant. Thus, one marker from each of the “mirror” pairs was arbitrarily discarded using the *drop.markers()* function, retaining the second group with phased markers. The smaller linkage groups that were not part of the 12 main mirror linkage group pairs were also removed.

### Creating a linkage map

Markers were reordered using the *orderMarkers()* function with parameters *window = 7, error.prob* = 0.0001, *map.function* = “haldane”, *sex.sp* = F. The *orderMarker()* function uses an algorithm that locally orders markers to minimize the number of crossovers. The window parameter value corresponds to the number of markers included in a sliding window of permuted markers. If multiple arrangements result in the same number of crossovers, one is chosen at random. Thus, it is expected that the result from each *orderMarker()* will be slightly different from the next. To deal with this, we ran the marker order procedure 9 separate times until they passed the below visual inspection.

Marker order was visually assessed using the combined heatmap of pairwise RF and LOD scores generated by the *plot. RF()* function. For each linkage group, the following criteria were used to determine if the marker placement across linkage groups and order within the linkage group was acceptable: (i**)** Marker placement: markers within their linkage group have low pairwise RF and high LOD (yellow) but high pairwise RF and low LOD (blue) outside their linkage group. (ii**)** Marker order: within the linkage group comparison block, there is a solid yellow line from the bottom left to top right for each.

If the criteria was not met, then markers for that specific linkage group were reordered using the same function and parameters until the above criteria is met. Supplementary Figure S2 illustrates this process, with panels A and B shown as examples of heat maps before and after marker ordering, respectively.

With so many markers, the RF/LOD heat map visual diagnostic alone is not sufficient to identify the best result. Therefore, for each linkage group, we used the *compareorder()* function using the parameter *error.prob* = 0.0001 to compare the likelihood of the marker orders and chose the order with the highest overall LOD score. Finally, genetic distance between markers was estimated using the *est.map()* function with default parameters.

### Map assessment

To visualize the contributions from the 12 main scaffolds and the unintegrated contigs to the linkage groups, we plotted the linkage map by linkage group and location (cM). The 12 main scaffolds were uniquely colored while the unintegrated contigs were all colored in black (Figure 3). The *countXO()* function was used to remove any individual with an unexpected number of crossovers. Comparative analysis between linkage and physical maps was accomplished by plotting genetic distance in cM to the physical distance in Mbp for each linkage group and corresponding scaffold. The *geno.table()* function was used to identify loci affected by segregation distortion. The chi-square test *P*-value threshold was Bonferroni adjusted accounting for the final number of markers (0.05/1919).

## Results

### Sequence capture efficiently target desired SNP positions

To genotype this S1 population (439 individuals), we selected 4884 positions that were heterozygous in CMEN13, as SNP markers, corresponding to a density of approximately 1 SNP per 100 kb throughout the genome (see Materials and Methods). Specifically, 4846 of these SNPs covered the 12 main scaffolds, while the remaining 38 SNPs targeted some of the unintegrated contigs (Table 1). The remaining scaffolds, representing _**∼**_0.96% of the genome size, were not targeted because valid SNP positions could not be identified.

**Figure 2:**
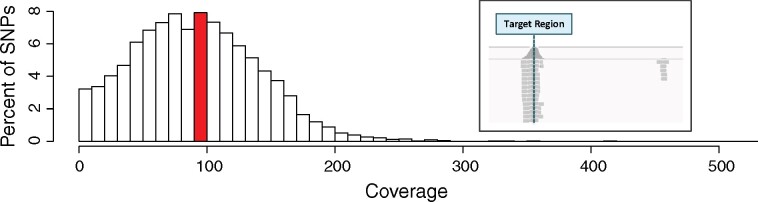
Distribution of sequence coverage per targeted SNP position. Extreme coverages above 500X are not shown. For each targeted SNP position, the average coverage was calculated across all libraries. The mean coverage per targeted SNP was 96X (highlighted in red), with ∼93% of SNPs falling within the acceptable coverage thresholds (20–1000X coverage). Average coverage of the target regions, including the entire sequences covered by the probes, was 90X, while the average coverage of the nontargeted space was 0.44X, corresponding to a 203-fold enrichment of the target sequences. (Inset) Typical Integrative Genomics Viewer (IGV) image of reads mapping to a targeted SNP. As expected, the captured SNP (denoted at a dashed vertical line) is covered by many reads, while few reads map off-target.

For each individual, a genomic Illumina library was constructed and subjected to sequence capture targeting the selected SNP positions, and sequenced. After the raw sequencing reads were demultiplexed and trimmed, a total of _**∼**_940 million reads were retained, or _**∼**_2.14 million reads per individual (Figure 1). After alignment to the draft reference genome, and removal of clonal reads, _**∼**_1.5 million reads per individual remained. With the remaining reads, average coverage at each targeted SNP was 96X (Figure 2). SNPs with extreme mean coverage values were removed, leaving _**∼**_93% of the SNPs exhibiting the acceptable coverage threshold (between 20-1000X).

To assess capture efficiency, we estimated fold-enrichment of targeted sequences compared to the rest of the genome in our sequencing reads and observed a 203-fold enrichment of the targeted regions (90X mean coverage) compared to the rest of the genome (0.44X mean coverage). To corroborate this measurement, we visually inspected mapped reads to evaluate specificity to on-target regions (Figure 2). As expected, the bulk of the reads centered at targeted SNP positions, and tapered off on either side. Reads only mapped to nontargeted sites sporadically.

### Sequence capture enables high-quality genotyping

To minimize genotyping errors, genotype calls that were ambiguous (less than 20X coverage), or suspicious (more than 1000X coverage) were discarded. Markers for which more than 20% of the genotype calls did not meet these coverage thresholds were removed, leaving 4657 markers across 439 individuals.

Further filtering was applied by tallying the number of markers genotyped for each individual, and the number of individuals typed for each marker (Supplementary Figure S1B). Specifically, markers with less than 300 individuals genotyped (586 markers), and individuals with less than 3500 markers genotyped were filtered out (19 individuals). Finally, individuals suspected of being double-sampled were removed, using a threshold of 90% similarity. We identified 5 pairs of individuals at this threshold and removed one individual from each pair (Supplementary Figure S1C).

Often, markers displaying segregation distortion are discarded when building an initial map. However, preliminary analysis showed that some scaffolds were predominantly composed of distorted markers. Thus, we decided to keep the distorted markers because discarding them would compromise our ability to construct a map. However, markers with more than 90% heterozygous calls were removed. With this filter, we further removed 29 markers. At this point, 4042 high-quality markers across 415 individuals remained.

Finally, tightly linked markers exhibiting identical genotypes in all considered individuals are redundant. For this reason, within a group of tightly linked markers, the marker with the least amount of missing data was retained at random. A total of 1905 redundant markers were removed, leaving a total of 2137 informative markers across 415 individuals for linkage analysis. In this final set, the mean number of genotyped individuals per marker was 402 (97% of the individuals), and the mean number of genotyped markers per individual was 2071 (97% of the markers). The overall final percentage of missing data was 3.1%.

### Dense genotype data allows for the creation of a dense, haplotype-phased, linkage map

First, we needed to phase the markers of the dataset, which was performed following a method previously described (Gadau *et al.* 2001). Working with unphased data are problematic because unphased adjacent SNPs appear unlinked. To resolve this issue, we introduced into the dataset a set of artificial “mirror” markers, with the complementary genotype phase for each marker. Considering that each marker was duplicated in this strategy, we started the linkage group analysis with 2137 “mirror” marker pairs, or 4274 total markers. We were able to assign 1919 of these marker pairs to 12 linkage group pairs (corresponding to the 12 expected chromosomes), in which the markers for each linkage group had been phased. The remaining 218 mirror marker pairs went into separate linkage groups, with at most 7 markers each, and were dropped (these were considered unlinked to main chromosomes). Finally, we arbitrarily picked one from each pair for linkage map construction.

While we do not have prior information regarding the expected number of crossovers per chromosome for *M. suaveolens* specifically, an acceptable range based on other plant species is 1–3 crossovers per chromosome per meiosis (Mercier *et al.* 2015). The number of crossovers was within expected range for all 415 individuals, with the frequency of distribution ranging from 7 to 35, averaging 18.

**Figure 3:**
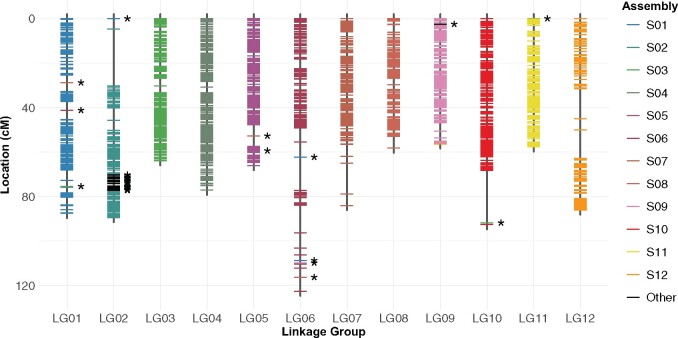
Final linkage map. Markers from each of the 12 main physical scaffolds are color-coded. Markers from smaller unintegrated contigs are colored in black and highlighted with asterisks. Genetic distance was calculated using the Haldane mapping function.

The final map consisted of 1919 phased SNPs mapped to 12 linkage groups. The total length of the linkage map was 942.17 cM, with linkage groups ranging from 56.32 to 122.61 cM and averaging to 78.51 cM (Table 2). The average distance between markers was 0.5 cM across 12 linkage groups, but 7 marker pairs in LG02, LG06, LG07, LG10, and LG12 were >10 cM apart (Figure 3). The largest gap (25.53 cM) is found at the distal end of LG02. Some of these gaps are associated with the reassignment of markers from the original scaffold to the different linkage groups, or assignment of markers from unintegrated contigs to LG02 (23 markers) and LG09 (1 marker).

**Table 2:** Final linkage groups and marker statistics

Linkage group	No. markers	From different scaffold	From unintegrated contig	Marker density (cM)	Length (cM)	Largest gap (cM)	Gaps larger than 10 cM	No. distorted markers	Distorted markers (%)
LG01	168	3	0	0.52	87.65	4.78	0	168	100.00
LG02	165	1	23	0.54	89.48	25.53	1	3	1.82
LG03	191	0	0	0.33	63.96	3.34	0	2	1.05
LG04	210	0	0	0.37	77.20	2.54	0	0	0.00
LG05	176	2	0	0.38	66.11	4.97	0	92	52.27
LG06	140	4	0	0.88	122.61	15.00	2	11	7.86
LG07	146	0	0	0.58	84.09	13.83	1	78	53.42
LG08	133	0	0	0.44	58.26	5.32	0	1	0.75
LG09	125	0	1	0.45	56.32	3.67	0	1	0.80
LG10	195	1	0	0.48	92.64	23.67	1	5	2.56
LG11	140	1	0	0.41	57.72	2.74	0	2	1.43
LG12	130	0	0	0.66	86.14	13.56	2	2	1.54

The final genetic map contains 1,919 markers, spanning a total of 942.17 cM.

### Comparative analysis between linkage and physical maps

**Figure 4:**
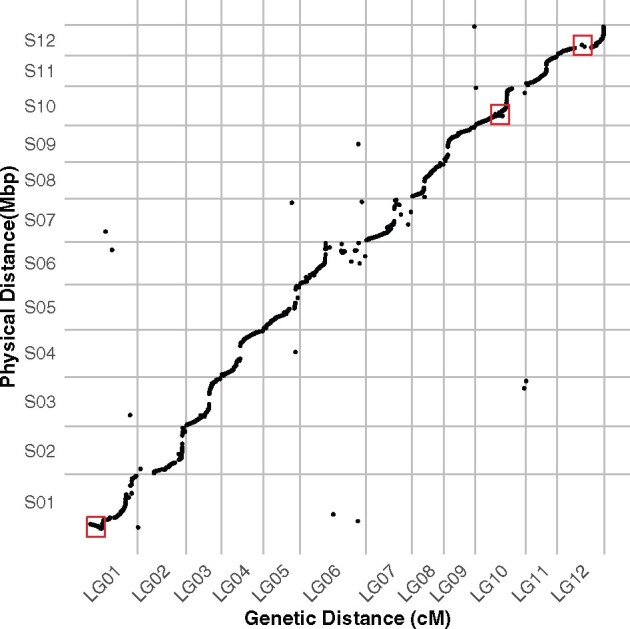
Comparative analysis of the final linkage groups and assembly. Marey map with genetic distance in cM represented on the *x*-axis, and the physical distance in Mbp represented on the *y*-axis. Examples of inversions are highlighted with red rectangles.

To evaluate the quality of the draft genome assembly, we compared the linkage and physical maps. Specifically, we investigated whether markers from the same scaffold were assigned together to a linkage group, and evaluated if the marker order was in agreement between scaffolds and linkage groups. Collinearity is indicated by a sigmoidal relationship between the physical and genetic distance (Figure 4). The near vertical region of the sigmoid is marked by a coldspot, characterized by large physical distance and small genetic distance, and probably indicative of heterochromatic regions. Overall, we observed high synteny as the majority of the markers mapped within their expected linkage groups, with no more than 4 markers assigned to a different scaffold for each linkage group. Some discrepancies, such as notable inversions, are visible (red boxes in Figure 4). Furthermore, markers at one end of LG06 and LG07 were heavily reorganized.

### Our genetic map reveals signs of strong segregation distortion

**Figure 5:**
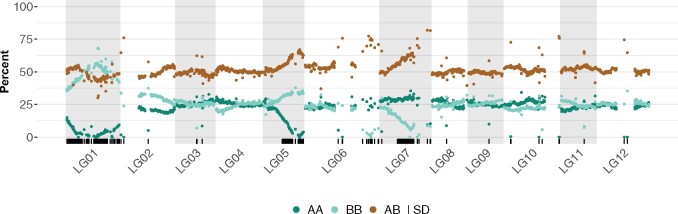
Segregation distortion in the S1 population. Percentage of AA, BB, and AB calls for each SNP marker are represented in dark green, light green, and brown, respectively. Markers exhibiting segregation distortion based on a chi-square test with Bonferroni adjusted *P*-value are marked as ticks on the bottom track.

With the S1 mapping population, we expect allele types AA, AB, and BB to be observed 25, 50, and 25% of the time, respectively. To detect segregation distortion, we compared these expected percentages to the observed percentage at each marker. Significantly distorted markers were identified using a Bonferroni corrected chi-square test. Most linkage groups were not affected by distortion (Table 2) with three notable exceptions: (i**)** the entire length of LG01, (ii**)** the distal end of LG05 and LG07, (iii**)** markers placed inside linkage map gaps, as in LG02, LG06, LG07, LG10, and LG12 (Figure 5). Most cases of distortion favored the heterozygous state or homozygous state from one parent *vs* the other. Segregation distortion can be indicative of artifacts in the case of more isolated markers, but can also be indicative of specific selection in the case of larger sets of consecutive markers, as observed here in several linkage groups.

## Discussion

We have constructed the first high-density linkage map of apple mint (*M. suaveolens*), from 1919 high-quality SNPs mapped to 12 linkage groups, which correspond to the expected 12 chromosomes of mint (Table 1). The map length is 942.17 cM, with an average of 78.51 cM per linkage group. Average marker intervals were 0.5 cM with some notable gaps greater than 10 cM. Overall, the linkage map shares a lot of synteny and collinearity with the physical map (Figure 4), confirming the quality of the physical assembly. Furthermore, we were able to incorporate 24 markers from four contigs that had been left unincorporated in the physical map. We found a few discrepancies between the physical and linkage maps, such as possible inversions and translocations, suggesting areas where the physical assembly can be improved (Figure 4). In this study, 24% of the markers used for the linkage map exhibited segregation distortion. Most of these are found in LG01, where all markers were affected, and LG05 and LG07, where almost half a section of the linkage group contained markers that were distorted (Figure 5).

Low sequence coverage, and the presence of missing data in mapping datasets can cause problems in assessing the true recombination rate between markers and thus, lead to miscalculated genetic distances or worse, misplacement of markers when creating genetic maps. Furthermore, markers with excess missing data provide little power toward finding linkage among the scaffolds. Sequence capture addresses these issues by targeting specific markers and yielding localized high coverage. This reduces the incidence of missing data, and because of its specificity, reduces the overall sequencing cost per informative marker. We demonstrate this by successfully targeting specific and informative sites across the genome. The high specificity of our targeting yielded an average SNP coverage of 96X, with a 203-fold enrichment across the targeted space compared to the rest of the genome. This high coverage provided a reliable dataset and after stringent filtering of markers, we were able to input 2137 markers into genetic map construction. These remaining markers only had 3.1% missing data, making imputation of missing marker genotypes unnecessary.

These results are comparable to those obtained previously in other species. For example, Tennessen *et al.* were able to construct a dense linkage map of diploid strawberry using a single selfed individual and sequence capture (Tennessen *et al.* 2014). Similarly, high-density linkage maps were obtained using sequence capture in durum wheat (Holtz *et al.* 2016) and loblolly pine (Neves *et al.* 2014), with similar numbers of markers, coverage, and low percentage of missing data. Previous reports also showed that this platform could also be successfully applied to polyploid strawberries (Tennessen *et al.* 2014). Moreover, capture probes allow for a relatively high percentage of divergence between probe sequence and captured sequence, allowing for the successful capture of homologous sequences from related species, and enabling evolutionary studies as well (Jones and Good 2016). Together, these suggest that this approach is likely to be successful for allopolyploid spearmint and peppermint as well.

In our experiment, the total genomic space directly targeted was only _**∼**_0.13% of the genome. This allowed us to achieve high coverage over these targeted regions at very low cost. In our case, sequencing of our 500 diploid mint clones using whole-genome sequencing and a 50X coverage would have cost _**∼**_$12,500, while the combined cost of the capture probe synthesis (smallest custom capture set from Arbor Biosciences) and sequencing of the captured libraries amounted to a total of _**∼**_$7,000. As for any large-scale experiment, the cost-benefit analysis greatly depends on the specifics of the experiments though. As a rule, the larger the number of individuals, and the smaller the target space, the more cost-effective the capture becomes, since the cost of probe design and capture probe synthesis are fixed. In our case, we could have further decreased the cost of sequencing because we targeted too many SNPs for the size of our population. Therefore, many markers were redundant and we only retained approximately a third of our original marker set. The discarded markers would be useful for eventual future fine-mapping but we could also have started with fewer targeted SNPs and further limited sequencing costs.

With 420 typed individuals, we also have the power to confidently identify loci affected by segregation distortion. There were occasionally distorted markers throughout all linkage groups (<10%), some of which corresponded to apparently inflated genetic distances between marker pairs. In cases where distorted markers occur in clusters, the distortion is likely due to biological factors rather than technical issues. However, if these are isolated markers, it indicates mis-scoring of markers and should be removed from the map. Further evaluation of these markers will be needed to decide if their placement is reasonable. The possibility that these correspond to repeated, polymorphic regions could be investigated by comparing observed and expected allele ratios. Deviations could suggest the presence of copy number variation and the subsequent removal of these markers.

Segregation distortion is very common in mapping populations (Li *et al.* 2010) (Taylor and Ingvarsson 2003) and distorted loci have the potential to complicate map construction. Removing them can be simpler, but comes with the disadvantage of potentially excluding large segments of the genome, and may not be beneficial. Indeed, previous modeling of the effect of including distorted markers suggested that they did not significantly affect marker order or map length (Hackett and Broadfoot 2003). Recently, a study in soybean looked at the effect of distorted markers on the construction of a high-density linkage map (Zuo *et al.* 2019) close in size to the one presented here. They concluded that including distorted markers resulted in genetic maps that were in better agreement with the genome, including more markers, and did not affect marker grouping (Zuo *et al.* 2019).

Strikingly, in our map, all markers in LG01, as well as _**∼**_50% of adjacent markers in LG05 and LG07, exhibited segregation distortion (Figure 5). The allelic ratios observed at those regions are each consistent with a different scenario. For LG01, the observed _**∼**_50% of BB and AB genotypes, combined with the almost complete absence of AA genotypes, suggest a strong selection against homozygous A genotypes at the zygotic level, but not at the gametic level, possibly linked to a lethal recessive allele. On the other hand, for LG05 and LG07, we observed _**∼**_66% AB genotypes, and _**∼**_33% BB or AA genotypes, respectively. This situation suggests a strong gametic selection against one specific haplotype (selection against A gametes for LG05 and selection against B gametes for LG07). In all three of these cases, understanding the mechanisms underlying these trends would possibly provide an approach to improving fertility in this species.

Genetic maps remain one of the most versatile and powerful genetic tools (Fierst 2015). In addition to the traditional uses of genetic maps, they now provide unique validation tools for genome assemblies (Yu *et al.* 2019). Specifically, dense genetic maps such as those that can be obtained from whole-genome sequencing data, can assist in identifying large-scale assembly errors such as inversions or translocation, help integrate unanchored contigs, and reach chromosome-level assemblies. More importantly, by virtue of the fact that they are built from recombination information, they provide independent linkage information. Finally, as in this study, genetic maps can provide haplotype information that can be instrumental in many downstream functional genetic analyses.

The map produced here provided independent data to assist the assembly of a high-value genome. While the linkage and physical maps were generally in agreement, we were able to use the information generated from the linkage map to correct the physical assembly. Comparative analysis between the linkage and physical maps also provided useful insights into the genomic features of the *M. suaveolens* genome. For example, the physical sequence assembly method produced chromosomal level scaffolds but no information on haplotype phasing. With our capture dataset, we were able to derive phased haplotypes easily. In addition, we were able to identify regions with low recombination (coldspots), which could correspond to centromeric regions, and regions of high recombination (hotspots).

Our results demonstrated the value of the capture-based genotyping method for the rapid generation of high-density linkage maps. The development of a genome assembly for the ancestral diploid, *M. suaveolens*, will provide an invaluable new genomic resource for the breeding of mint cultivars.

## Supplementary Material

jkab232_Supplementary_Data
