# Supplementary material for: Efficient construction of a linkage map and haplotypes for Mentha suaveolens using sequence capture
Source: G3 (Bethesda). 2021 Jul 14;11(9):jkab232. doi: 10.1093/g3journal/jkab232 (PMC8496254; doi:10.1093/g3journal/jkab232)
Supplement: jkab232_Supplementary_Data [file jkab232_supplementary_data.zip › jkab232-suppl_data/GENETICS-G3-2021-402261-s01.pdf]

SUPPLEMENTAL INFORMATION

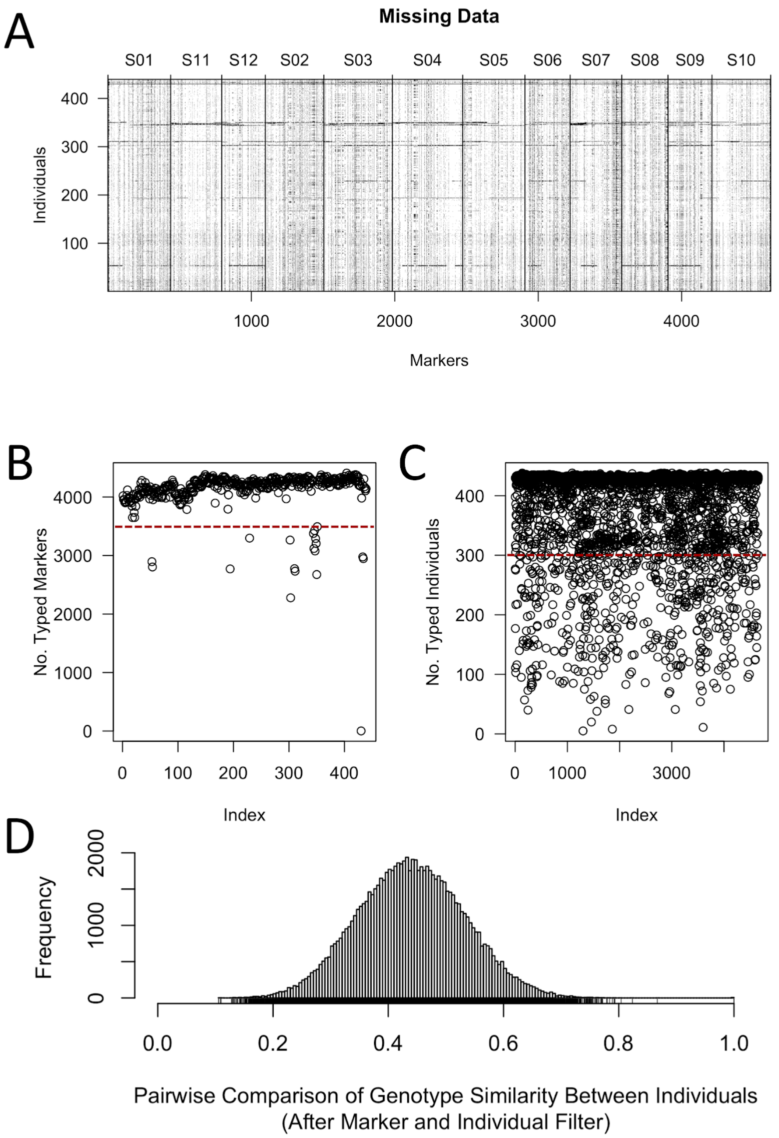

Figure S1 Markers and individuals filtering steps
(A) Plot of missing genotype data (indicated by black pixels) for each marker (x-axis) and each individual (y-axis). **(B)** Number of markers with genotyping data, for each individual. Individuals with less than 3,500 data points were discarded (red dotted line). **(C)** Number of individuals with genotyping data, for each marker. Markers for which data was available from less than 300 individuals (75%) were discarded (red dotted line). **(D)** Distribution of the proportion of identical genotypes after filtering for missing data, as shown in B and C. At the threshold of 90% identity, 5 pairs of individuals were identified and only one individual was retained for each pair.

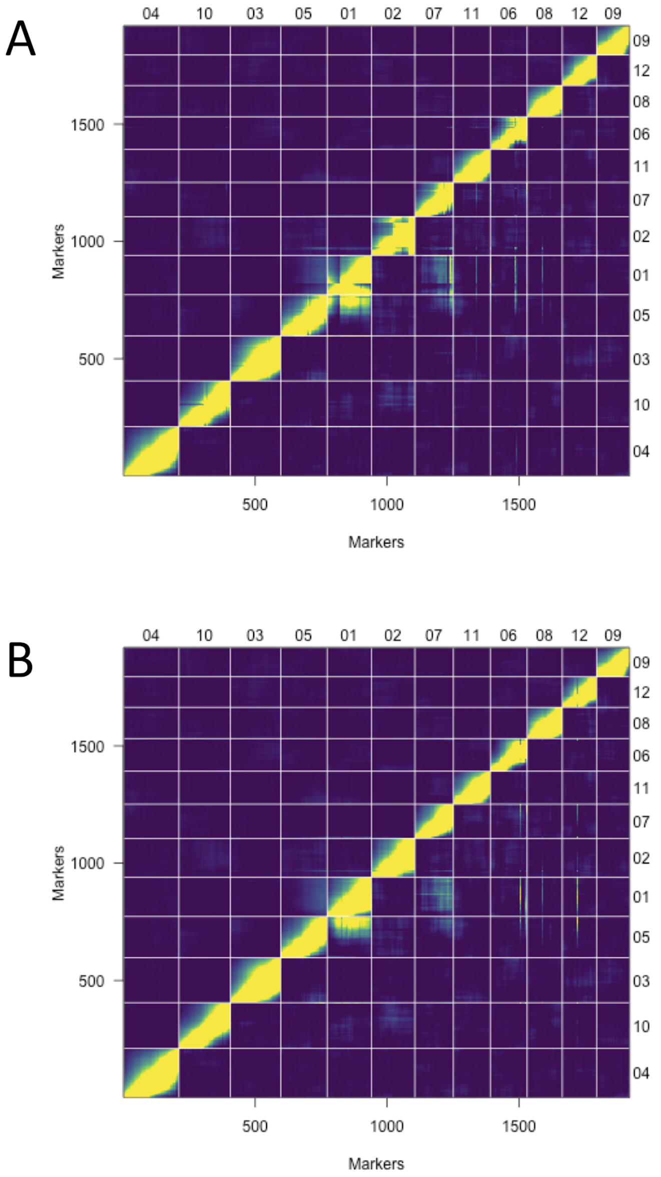

Figure S2 Heat map of the pairwise recombination frequency (RF) (upper left triangle) and corresponding LOD (lower right triangle) for all pairs of markers. (**A)** Heat map of the 12 linkage groups prior to marker ordering. (**B)** Heat map of the final 12 linkage groups after markers were ordered.

Type	Assembly Name	Size (Mbp)	Genome Covered (%)	Starting SNPs	Filter					
					Coverage	Excessive Missing Data	Potential Duplicates	Excessive Heterozygotes	Redundant	Unlinked
**Scaffold**	S01	62.45	11.64	464	437	374	374	372	196	168
	S02	51.27	9.56	430	408	338	338	338	176	141
	S03	51.75	9.65	491	478	418	418	415	214	194
	S04	49.09	9.15	510	490	453	453	449	223	211
	S05	46.64	8.70	459	434	396	396	395	189	174
	S06	44.69	8.33	323	315	265	265	263	160	137
	S07	44.57	8.31	375	360	290	290	284	166	149
	S08	38.13	7.11	335	322	272	272	269	158	133
	S09	37.15	6.93	328	307	264	264	261	143	125
	S10	40.48	7.55	428	409	365	365	364	201	194
	S11	31.60	5.89	381	356	327	327	326	148	139
	S12	30.25	5.64	322	304	277	277	275	139	130
**Contig**	X1059	0.02	0.004	1	1	1	1	0	0	0
	X1121	0.01	0.003	1	1	1	1	1	0	0
	X1124	0.01	0.003	1	1	0	0	0	0	0
	X1181	0.01	0.001	1	1	1	1	1	1	1
	X233	0.12	0.023	1	1	1	1	1	0	0
	X321	0.45	0.084	1	1	0	0	0	0	0
	X368	0.05	0.009	1	1	1	1	1	0	0
	X394_1	1.35	0.251	22	21	20	20	20	18	18
	X394_2	0.94	0.175	6	6	6	6	6	4	4
	X394_3	0.12	0.023	2	2	1	1	1	1	1
	X713	0.01	0.001	1	1	0	0	0	0	0
**GRAND TOTAL**				4884	4657	4071	4071	4042	2137	1919

Table S1 Marker remaining after each filtering step

For each pseudomolecule, the starting number of SNP markers (SNP List), remaining markers after the filter based on sequence coverage (Coverage), remaining markers after markers with excess missing information were discarded (Excessive Missing Data), remaining markers after the removal of one of a pair of individuals with >90% identical genotype calls across markers (Potential Duplicates), remaining markers after the removal of markers with >90% heterozygous calls (Excessive Heterozygotes), remaining markers after removal of redundant markers (Redundant), and remaining after the removal of unlinked markers (Unlinked) are indicated.
